# Elevated triglyceride-glucose (TyG) index predicts impaired islet β-cell function: A hospital-based cross-sectional study

**DOI:** 10.3389/fendo.2022.973655

**Published:** 2022-09-30

**Authors:** Zi Chen, Jie Wen

**Affiliations:** ^1^ Department of Health Management, The Third Xiangya Hospital, Central South University, Changsha, China; ^2^ National Clinical Research Center for Metabolic Diseases, Metabolic Syndrome Research Center, Key Laboratory of Diabetes Immunology, Ministry of Education, and Department of Metabolism and Endocrinology, The Second Xiangya Hospital of Central South University, Changsha, China

**Keywords:** TyG index, Islet β-cell function, Insulin secretion, T2DM, Cross-sectional studies

## Abstract

**Objective:**

To explore the relationship between the TyG index and the insulin secretion function of pancreatic β-cells, and to determine the possibility of the TyG index in predicting β-cell dysfunction and the development of diabetes.

**Methods:**

A cross-sectional study was performed among 914 participants who took their annual health checkups at the Third Xiangya Hospital. The early- and late-phase pancreatic β-cell secretion was assessed based on the results of the oral glucose tolerance test (OGTT). In addition to anthropometric parameters and laboratory parameters, information about health-related habits and disease histories was obtained from the National Physical Examination Questionnaire. Partial correlation analysis was used to study the relationship between the TyG index and pancreatic β-cell function. The receiver operating characteristic (ROC) curve was used to calculate the cut-off points of the TyG index in predicting β-cell dysfunction. According to the OGTT results and medical history, the participants were categorized into three groups: the normal glucose tolerance group (NGT, n=276), the impaired glucose regulation group (IGT, n=323), and the diabetes group (DM, n=315). The correlation between the TyG index and β-cell function among the three groups and the association between the TyG index and glucose metabolic conditions were further explored.

**Results:**

The TyG index was negatively correlated with the indexes that reflect the early and late secretory function of β-cells, not only in the NGT group but also in the IGT and DM group. The minimum cut-off values for the TyG index to identify the risk of early- and late-phase β-cell dysfunction are 9.08 and 9.2 respectively. The TyG indexes of the IGT and DM group were higher than that of the NGT group, and with the growth of the TyG index, the risk of prediabetes and diabetes increased significantly.

**Conclusion:**

Increased TyG index is associated with impaired β-cell function regardless of the glucose metabolic conditions. The TyG index is an alternative indicator for predicting β-cell dysfunction.

## Introduction

Type 2 diabetes mellitus (T2DM) has become a worldwide health problem in recent years, which influences around 8% of people all over the world (1). Of note, the prevalence of T2DM dramatically increased during the latest decades in China (2), which makes China become one of the epicenters of this epidemic. The two major underlying mechanisms in the pathogenesis of T2DM are insulin resistance and deficient insulin secretion (3). Previous researches indicate early functional defects in β cells already exist in pre-diabetes (4, 5). Meanwhile, accumulated evidence suggests the improvement of β-cell function by lifestyle intervention or medical treatment could effectively prevent the development of T2DM (6). Due to the critical role of β cell dysfunction in the progression and prognosis of T2DM, it’s crucial to identify an indicator that could detect and assess the β cell function at the very early stage.

In response to glucose stimulation, β cells showed dynamic and biphasic insulin secretions, which are first- and second-phase secretions (7). The first-phase insulin secretion (FPIS) is an acute response of β cells to secret insulin when exposed to intravenous glucose within 10 min or oral glucose within 30 min. After that, a gradual and moderate increase in insulin level is defined as the second-phase insulin secretion (SPIS) when persistent stimuli exist (8). These two phases of insulin secretion have distinct functions. For instance, the FPIS is more important for inhibiting endogenous glucose synthesis and downregulating blood glucose after meals (9). The observation of insulin secretion is essential for β-cell function assessment. At present, the well-known methods for evaluating β-cell function are the high-glucose clamp test (HGCT), intravenous glucose tolerance test (IVGTT), oral glucose tolerance test (OGTT), and homeostasis model assessment (HOMA) β index (10, 11). Although the HGCT is considered the golden standard for quantification of insulin secretion, its clinical application was limited due to the complicated and cumbersome processes. In addition, HGCT is only suitable for the assessment of islet function in people with normal glucose tolerance. The IVGTT and OGTT methods require several venous blood collections, which require a long-time test and cost amounts of money. The shortcoming of the HOMA-β index is it may not accurately reflect the absolute β-cell function due to this index being calculated from the basic concentrations of fasting plasma glucose and insulin but not from dynamic values in response to insulinogenic stimuli (12). Insulin resistance also affects the assessment of islet function, and the HOMA-β index does not take insulin resistance into account. Hence, it’s urgent to explore a biomarker that could predict the β-cell dysfunction in a convenient, economic, and reliable way.

Triglyceride-glucose index (TyG) was calculated from the serum triglycerides (TG) and fasting plasma glucose (FPG) levels, which have been reported to be associated with several chronic diseases, for instance, cardiovascular disease, metabolic fatty liver diseases, and metabolic syndrome (13–16). Recent studies uncovered a correlation between the TyG index and insulin resistance (17, 18). However, it’s unclear whether TyG is associated with the β-cells insulin secretion function. Since lipotoxicity and glucotoxicity are two major components to induce β-cell dedifferentiation and apoptosis, and subsequently β-cell dysfunction (19, 20). We are curious about the relationship between the TyG index and the insulin secretion of pancreatic β cells. In this study, we analyzed the association between TyG and Homa-β and early/late–phase indices based on OGTT. We also assessed the TyG index in people with different statuses of glucose metabolic disorders. This study revealed the TyG index is negatively associated with β-cell function, which uncover a novel indicator to predict β-cell dysfunction at a very early stage.

## Subjects and methods

### Research subjects

**Figure 1:**
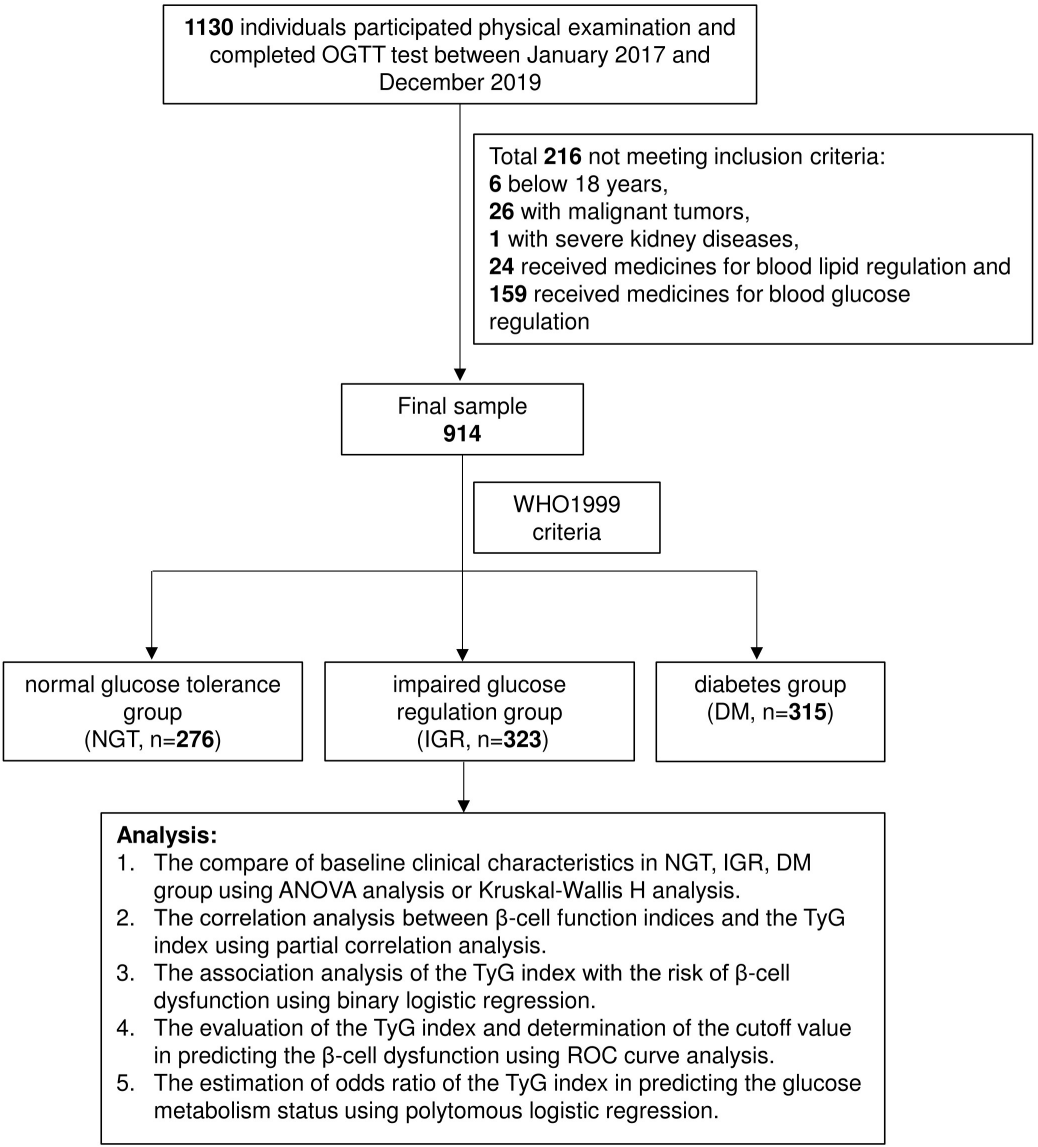
Flow chart of the study design. A total of 1130 individuals were recruited for this study at the beginning. Among them, 216 individuals that not match the criteria were excluded. And the left participants were divided into 3 subgroups (NGT: n=268, IGT: n=352, DM: n=314) by the WHO 1999 diagnostic criteria.

The research subjects of this study were recruited from physical examinations at the Third Xiangya Hospital from Jan 2017 to Dec 2019. All the participants signed the written informed consent form, and this study was approved by the Ethical Committee of the Third Xiangya Hospital of Central South University (Ethics Board Approval Number: 22093). All the subjects are over 18 years old and accepted OGTT. Subjects with severe acute and chronic inflammation, with a history of severe cardiovascular, kidney, or liver diseases, with malignant tumors, or who received medicines for blood glucose or blood lipid regulation were excluded from the study. We finally enrolled 914 subjects (male: n = 665, female: n = 249) aged from 18-65 years old for this study. The flow chart of this study was shown in **Figure 1**.

### Criteria for subgrouping

Normal glucose tolerance (NGT) is defined as 3.9 mmol/L ≤ Fasting plasma glucose (FPG)< 6.1mmol/L, and 2-hour plasma glucose level following OGTT (2-h OGTT) 7.8mmol/L. Impaired glucose tolerance (IGT) as 6.1 mmol/L ≤ FPG< 7.0mmol/L or 7.8mmol/L ≤ 2-h OGTT< 11.1mmol/L. DM patients were defined as FPG ≥ 7.0mmol/L or random plasma glucose ≥ 11.1mmol/L or OGTT 2-h OGTT ≥ 11.1mmol/L (21).

### Calculation formulas for pancreatic β-cell function

The calculation of indexes representing pancreatic β-cell function came from the results of OGTT. The formula for each index was listed below:

(1) Homa−β=(20*I0)/(G0−3.5);

(2) Early-phase BCF indices (22–26): I30/I0 Ratio = I30/I0,

CP30/ CP0 ratio= CP30/ CP0

△I30/△G30 ratio= ( I30 − I0)/( G30− G0) ,

△CP30/△G30 ratio= (CP30 − CP0)/( G30− G0) ,

CIR30x10−2 = I30/[G30 ×(G30 −3.89)] ,

Stumvoll first phase x10−2= 1283+(1.829×I30)−(138. 7×G30)+(3.772×I0) ,

(3) Late-phase BCF indices (23–26):

I120 /I0 ratio= I120/I0 ,

CP120 /CP0 ratio= CP120/ CP0,

        △I120 /△G120 ratio= (I120 − I0)/( G120− G0) ,


        △CP120/△G120 ratio= (CP120 − CP0)/( G120− G0)

        CIR120x10−2= I120/[G120 ×(G120 −3.89)] ,

        Stumvoll second phase x10−2= 287 + (0.4164× I30) − (26.07×G30)+(0.9226×I0) ;

(4)  Matsuda index =10000/(G0 × I0 × Gmean × Imean) 0.5 (27);

(5)  Disposition index (DI)=BCF indices* Matsuda index;

(6)  The TyG index= ln[TG (mg/dL)×FBP (mg/dL)/2] (28).

### Measurement of serum biochemical indices

The biochemical indices were detected in the Endocrine Laboratory of the Third Xiangya Hospital. All the participants were subjected to an OGTT test according to WHO guidelines, and plasma glucose, insulin, and C-peptide values were determined 30 minutes and 120 minutes after an oral 75g glucose load (glycemia was detected by hexokinase method, blood insulin and C-peptide were detected by immunochemiluminometric assays).

### Statistical analysis

Data were analyzed by SPSS software (SPSS 18.0; IBMInc., Chicago, IL, USA). The normally distributed data are expressed as means ± standard deviation, and the skewed data were presented as median (interquartile range). The ANOVA analysis was used to compare the differences of normally distributed variates between subgroups, and Kruskal-Wallis H was used for the analysis of skewed variates. Partial correlation analysis was used to find out the correlation between insulin secretion and TyG levels. Receiver operative characteristics (ROC) were analyzed to determine cutoff values of the TyG index in predicting β-cell dysfunction. Binary logistic regression was performed to identify TyG index levels associated with the risk of β-cell dysfunction and glucose metabolic condition. And polytomous logistic regression was performed to estimate the ORs of each category of TyG with different glucose statuses. Covariates were selected as potential confounders which may influence the results based on established correlations or plausible biological relations, and a change in effect estimate of more than 10%. And the changes in estimate over 10% in **Table 3** were shown in **Supplementary Table 1**. The *P*-values lower than 0.05 were considered statistically significant.

## Results

### Baseline clinical characteristics

There’s a total of 914 subjects (NGT=276, IGT=323, DM=315) enrolled in this study. The gender showed no difference between subgroups. However, the glycosylated hemoglobin (HbA1c), Body mass index (BMI), systolic blood pressure (SBP), diastolic blood pressure (DBP), total cholesterol (TC), fasting plasma glucose (FPG), triglyceride (TG), and TyG index, fasting insulin (FINS), fasting C-peptide (FCP), 2 hours postprandial glucose (2hPG) were increased with the appear and worsen of glucose metabolic disorders (*P*< 0.01). In addition, the high-density lipoprotein (HDL), 2 hours postprandial insulin (2hPINS), and Matsuda index are significantly different in the subgroups (**Table 1**).

**Table 1:** Clinical characteristics of research subjects.

	NGT	IGR	DM	*P*
n	276	323	315	
male	192 (69.57%)	239 (73.99%)	234 (74.29%)	0.36
Age (years)	44.68 ± 9.30	48.46 ± 9.14	50.92 ± 9.47	<0.01
HBA1C (%)	5.46 ± 0.32	5.72 ± 0.54	7.03 ± 1.55	<0.01
BMI (kg/m2)	24.92 ± 3.49	25.82 ± 3.06	25.95 ± 3.46	<0.01
SBP (mmHg)	121.50 ± 14.30	125.70 ± 15.56	129.82 ± 15.00	<0.01
DBP (mmHg)	75.35 ± 11.39	78.10 ± 11.31	80.13 ± 10.44	<0.01
HDL (mmol/l)	1.32 ± 0.28	1.26 ± 0.30	1.24 ± 0.25	<0.01
LDL (mmol/l)	2.83 ± 0.79	2.72 ± 0.89	2.84 ± 0.89	0.20
TC (mmol/l)	4.99 ± 0.92	5.14 ± 0.95	5.30 ± 1.02	<0.01
TG (mmol/l)	1.39 (1.00-2.20)	1.89 (1.32-2.94)	2.06 (1.46-3.03)	<0.01
FPG (mmol/l)	5.47 (5.08-5.75)	6.02 (5.52-6.40)	7.52 (6.70-8.89)	<0.01
TyG index	8.76 ± 0.60	9.17 ± 0.69	9.54 ± 0.73	<0.01
FINS (Uu/ml)	6.77 (4.49-9.40)	7.53 (5.22-11.24)	8.30 (5.49-13.06)	<0.01
FCP (ng/ml)	1.88 ± 0.62	2.14 ± 0.70	2.31 ± 0.81	<0.01
2hPG (mmol/l)	6.40 (5.70-7.2)	8.70 (7.90-9.60)	14.4 (12.1-17.5)	<0.01
2hPINS (Uu/ml)	36.22 (23.91-54.70)	58.44 (38.72-92.83)	48.03 (29.64-79.53)	<0.01
Matsuda index	110.71 (79.56-161.87)	85.55 (59.26-122.39)	65.03 (44.62-102.38)	<0.01

### Correlation between the TyG index and β-cell function in all subjects

Partial correlation analysis indicated that the TyG index was negatively associated with I_30_/I0 Ratio DI (r = -0.127, *P* = 0.02), CP30/CP0 Ratio DI (r = -0.298, *P*< 0.001), CIR_30_X10^-2^ DI (r = -0.146, *P<* 0.001), Stumvoll First phase X10^-2^ DI (r = -0.152, *P<* 0.001) that reflects the early secretory function of islet β cells. Meanwhile, TyG index was negatively correlated with I_120_/I_0_ ratio DI (r = -0.217, *P*< 0.001), CP_120_/CP_0_ ratio DI (r = -0.338, *P*< 0.001), and Stumvoll second phase X10^-2^ (r = -0.271, *P*< 0.001), which reflected late secretory function of islet β cells (**Table 2**). These results suggest that β-cell dysfunction was significantly associated with the TyG index demonstrated by several indices that reflects the β-cell secretory function correlated with the TyG index after adjusting age, gender, and so on. (**Table 2**).

**Table 2:** Association between TyG index and β-cell function in all subjects.

BCF	TyG	r	*P-*value
Homa-β		0.063	0.139
**Early-phase BCF indices**			
I_30_/I_0_ Ratio DI		-0.127a	0.02
CP_30_/CP_0_ ratio DI		-0.298b	<0.001
△I_30_/△G_30_ ratio DI		0.012b	0.775
△CP_30_/△G_30_ ratio DI		0.006b	0.892
CIR_30_x10^-2^ DI		-0.146c	<0.001
Stumvoll first phase x10^-2^ DI		-0.152c	<0.001
**Late-phase BCF indices**			
I_120_/I_0_ratio DI		-0.217d	<0.001
CP_120_/CP_0_ ratio DI		-0.338e	<0.001
△I_120_/△G_120_ ratio DI		-0.046f	0.269
△CP_120_/△G_120_ ratio DI		-0.041g	0.323
CIR_120_x10^-2^DI		-0.037c	0.380
Stumvoll second phase x10^-2^ DI		-0.271c	<0.001

A, Age, SBP, DBP, HbA1C, HDL, FINS, FCP adjusted; b, Age, SBP, DBP, HbA1C, HDL, FINS, FCP,TC adjusted; c, Gender, Age, SBP, DBP, HbA1C, HDL, FINS, FCP,TC adjusted; d, Gender, Age, SBP, DBP, HbA1C, FINS, FCP adjusted; e, Gender, Age, SBP, DBP, HDL, HbA1C, FINS, FCP adjusted; f, Age, SBP, DBP, HbA1C, FINS adjusted; g, SBP, DBP, HbA1C, FINS adjusted.

To further investigate the relationship between β-cell dysfunction and the TyG index. We defined the lower quartile of the early-phase or late-phase insulin secretion indices as β-cell dysfunction. Then we used binary logistic regression to obtain odds ratios (ORs) of β-cell dysfunction among participants with different TyG levels. After adjusting several covariates, the multivariable-adjusted (model 2) ORs among higher quartiles (Q2, Q3, Q4) are higher than the first quartile (Q1) for several early and late indices that reflect β-cell dysfunction, and the relative risk of β-cell dysfunction elevated with the increase of the TyG index from Q1 to Q4 (**Table 3**).

**Table 3:** Odds ratios for the risk of β cell dysfunction according to TyG quartiles.

β-Cell dysfunction indices	Quartiles of TyG	Model 1	Model 2
**Early-phaseβ-Cell dysfunction indices**			
I30/I0 Ratio DI		Reference	Reference
	Q1 (n = 232)	2.09 (1.13 to 3.86)	1.67 (0.57 to 4.94)
	Q2 (n = 229)	5.42 (3.07 to 9.57)	5.49 (2.01 to 14.98)
	Q3 (n = 232)	8.29 (4.74 to 14.50)	3.13 (1.09 to 9.00)
	Q4 (n = 221)		
	*P* for trend	<0.001	0.001a
CP30/CP0 ratio DI		Reference	Reference
	Q1 (n = 232)	2.14 (1.12 to 4.09)	1.86 (0.82 to 4.26)
	Q2 (n = 229)	5.83 (3.22 to 10.57)	4.92 (2.26 to 10.74)
	Q3 (n = 232)	10.55 (5.88 to 18.92)	9.59 (4.07 to 22.62)
	Q4 (n = 221)		
	*P* for trend	<0.001	<0.001b
△I30/△G30 ratio DI		Reference	Reference
	Q1 (n = 232)	2.61 (1.47 to 4.62)	2.38 (1.03 to 5.49)
	Q2 (n = 229)	4.33 (2.50 to 7.50)	2.96 (1.27 to 6.9)
	Q3 (n = 232)	6.47 (3.77 to 11.10)	2.45 (0.96 to 6.55)
	Q4 (n = 221)		
	*P* for trend	<0.001	0.09c
△CP30/△G30 ratio DI		Reference	Reference
	Q1 (n = 232)	1.69 (0.93 to 3.05)	1.32 (0.50 to 3.51)
	Q2 (n = 229)	4.64 (2.71 to 7.93)	3.25 (1.27 to 8.30)
	Q3 (n = 232)	6.35 (3.74 to 10.78)	2.18 (0.76 to 6.37)
	Q4 (n = 221)		
	*P* for trend	<0.001	0.02c
CIR30x10-2 DI		Reference	Reference
	Q1 (n = 232)	5.64 (2.57 to 12.33)	5.41 (1.63 to 17.96)
	Q2 (n = 229)	11.52 (5.39 to 24.62)	10.53 (3.19 to 34.79)
	Q3 (n = 232)	20.06 (9.46 to 42.54)	15.23 (4.08 to 56.81)
	Q4 (n = 221)		
	*P* for trend	<0.001	<0.01c
Stumvoll first phase x10-2 DI		Reference	Reference
	Q1 (n = 232)	2.32 (1.38 to 3.89)	1.50 (0.77 to 2.93)
	Q2 (n = 229)	2.44 (1.45 to 4.08)	1.32 (0.37 to 52.61)
	Q3 (n = 232)	4.69 (2.86 to 7.68)	1.93 (0.94 to 3.97)
	Q4 (n = 221)		
	*P* for trend	<0.001	0.31d
**Late-phase** **β-Cell dysfunction indices**			
I_120_/I_0_ratio DI		Reference	Reference
	Q1 (n = 232)	1.03 (0.61 to 1.76)	1.02 (0.56 to 1.86)
	Q2 (n = 229) Q3 (n = 232)	2.05 (1.26 to 3.32) 4.33 (2.73 to 6.86)	1.58 (0.90 to 2.78) 3.94 (2.21 to 7.02)
	Q4 (n = 221)		
	*P* for trend	<0.001	<0.001e
CP_120_/CP_0_ ratio DI		Reference	Reference
	Q1 (n = 232)	1.33 (0.75 to 2.34)	0.45 (0.15 to 1.32)
	Q2 (n = 229)	2.61 (1.55 to 4.40)	0.76 (0.28 to 2.09)
	Q3 (n = 232)	6.66 (4.06 to 10.94)	1.54 (0.49 to 4.85)
	Q4 (n = 221)		
	*P* for trend	<0.001	0.12f
△I_120_/△G_120_ ratio DI		Reference	Reference
	Q1 (n = 232)	0.72 (0.44 to 1.19)	0.53 (0.28 to 1.01)
	Q2 (n = 229)	1.36 (0.87 to 2.14)	0.89 (0.49 to 1.61)
	Q3 (n = 232)	2.61 (1.70 to 4.01)	1.19 (0.64 to 2.20)
	Q4 (n = 221)		
	*P* for trend	<0.001	0.07g
△CP_120_/△G_120_ ratio DI		Reference	Reference
	Q1 (n = 232)	0.72 (0.43 to 1.19)	0.49 (0.25 to 0.95)
	Q2 (n = 229)	1.40 (0.89 to 2.21)	0.98 (0.54 to 1.79)
	Q3 (n = 232)	2.64 (1.72 to 4.06)	1.42 (0.77 to 2.61)
	Q4 (n = 221)		
	*P* for trend	<0.001	0.01h
CIR_120_x10^-2^DI		Reference	Reference
	Q1 (n = 232)	2.98 (1.53 to 5.79)	2.10 (0.78 to 5.65)
	Q2 (n = 229)	6.51 (3.47 to 12.20)	4.60 (1.73to12.25)
	Q3 (n = 232)	12.50 (6.75 to 23.17)	7.77 (2.52to23.90)
	Q4 (n = 221)		
	*P* for trend	<0.001	<0.001c
Stumvoll second phase x10^-2^ DI		Reference	Reference
	Q1 (n = 232)	5.35 (2.70 to 10.58)	6.56 (2.29o18.86)
	Q2 (n = 229)	6.65 (3.39 to 13.05)	7.08 (2.48to20.22)
	Q3 (n = 232)	13.89 (7.19 to 26.86)	11.07 (3.76to32.55)
	Q4 (n = 221)		
	*P* for trend	<0.001	<0.001i

A, Age, SBP, DBP, HbA1C, FINS, FCP adjusted; b, Age, SBP, DBP, HbA1C,TC, HDL adjusted; c, Age, SBP, DBP, HbA1C,TC, HDL, FINS, FCP adjusted; d, Age, SBP, DBP, HbA1C,TC adjusted; e, Gender, HbA1C,TC adjusted; f, DBP, HbA1C, HDL, FINS, FCP adjusted; g, SBP, DBP, HbA1C adjusted; h, DBP, HbA1C adjusted; i, Age, Gender, SBP, DBP, HbA1C,TC adjusted.

### The cut-off value of the TyG index for predicting the β-cell dysfunction

**Table 4** showed the cut-off levels, the area under the ROC curve (AUC) values, sensitivity, specificity, and Youden index of TyG index in detecting the β-cell dysfunction. Among the early-phase β-cell dysfunction indices, CIR_30_x10^-2^ DI exhibited the highest AUC value (AUC = 0.74, 95% CI = 0.71-0.78, *P*< 0.01). The cut-off value for the highest Youden index (0.37) is 9.31. The sensitivity and specificity are 70% and 68%. the minimum cut-off point for predicting early-phase deficiency of islet β-cell function is 9.08. For the late-phase β-cell dysfunction indices, the AUC of CIR_120_x10^-2^ DI is the largest (AUC = 0.74, 95% CI, 0.70-0.77; *P*< 0.01). And the cut-off value is 9.2 for the highest Youden index (0.38). The sensitivity and specificity for this point are 76% and 62%. The minimum cut-off point for predicting late-phase β-cell function impairment is 9.2. Moreover, the AUCs were increased after adjusting the covariates (age, gender, and so on), which indicates the confounders adjusting increases the diagnostic accuracy of the TyG index in identifying people with β-cell dysfunction (**Supplementary Table 2**).

**Table 4:** Comparison of the ability of TyG to predict β-cell dysfunction.

Variable	AUC (95% CI)	Cut-off	Sensitivity		Specificity	Youden index	*P*-value
**Early-phase β-Cell dysfunction indices**							
I30/I0 Ratio DI	0.71 (0.67-0.75)	9.17	0.75	0.6		0.35	<0.001
CP30/CP0 ratio DI	0.73 (0.69-0.77)	9.31	0.71	0.68		0.39	<0.001
△I30/△G30 ratio DI	0.68 (0.64-0.72)	9.08	0.76	0.53		0.28	<0.001
△CP30/△G30 ratio DI	0.70 (0.66-0.74)	9.19	0.71	0.68		0.34	<0.001
CIR30x10-2 DI	0.74 (0.71-0.78)	9.31	0.7	0.68		0.37	<0.001
Stumvoll first phase x10-2 DI	0.65 (0.61-0.69)	9.36	0.55	0.67		0.22	<0.001
**Late-phase β-Cell dysfunction indices**							
I120/I0ratio DI	0.67 (0.63-0.72)	9.31	0.65	0.67		0.32	<0.001
CP120/CP0 ratio DI	0.71 (0.67-0.75)	9.31	0.7	0.69		0.38	<0.001
△I120/△G120 ratio DI	0.63 (0.58t-.67)	9.31	0.6	0.65		0.24	<0.001
△CP120/△G120 ratio DI	0.63 (0.58-0.67)	9.35	0.57	0.67		0.24	<0.001
CIR120x10-2DI	0.74 (0.70-0.77)	9.2	0.76	0.62		0.38	<0.001
Stumvoll second phase x10-2 DI	0.72 (0.68-0.75)	9.36	0.63	0.7		0.33	<0.001

### Association between TyG index and β-cell function in people with different glucose metabolic status

We classified the study population into three subgroups (NGT, IGT, and DM) by the status of glucose metabolism. And then we studied the correlation between the TyG index and β-cell function in these subgroups. Our results exhibited that the TyG index was negatively associated with I_30_/I_0_ Ratio DI (r = -0.282, *P*< 0.001), CP_30_/CP_0_ ratio DI (r = -0.414, *P*< 0.001), △CP30/△G30 ratio DI (r = -0.194, *P* = 0.003), CIR30x10-2 DI (r = -0.198, *P* = 0.002), I_120_/I_0_ ratio DI (r = -0.251, *P*< 0.001), CP_120_/CP_0_ ratio DI (r = -0.389, *P*< 0.001), CIR120x10-2 DI (r = -0.176, *P* = 0.007) and Stumvoll second phase x10-2 DI (r = -0.253, *P<* 0.001) in the IGT group. In the DM group, the TyG index is negatively correlated to I_30_/I_0_ Ratio DI (r = -0.218, *P* = 0.001), CP_30_/CP_0_ratio DI (r = -0.263, *P*< 0.001), △I_30_/△G_30_ ratio DI (r = -0.134, *P* = 0.033), △CP_30_/△G_30_ ratio DI (r = -0.161, *P* = 0.011), CIR_30_x10^-2^ DI (r = -0.265, *P*< 0.001), I_120_/I_0_ ratio DI (r = -0.247, *P*< 0.001), CP_120_/CP_0_ ratio DI (r = -0.282, *P<* 0.001), △I120/△G120 ratio DI (r = -0.144, *P* = 0.034), △CP_120_/△G_120_ ratio DI (r = -0.157, *P* = 0.024), CIR_120_x10^-2^ DI (r = -0.225, *P*< 0.001). In addition, we found the TyG index is also good at reflecting the insulin secretion function in people with normal glucose metabolism, demonstrated by a negative correlations exist between the TyG index and CP_30_/CP_0_ ratio DI, I_120_/I_0_ratio DI, CP_120_/CP_0_ ratio DI, and Stumvoll second phase x10^-2^ in NGT group (r = -0.218, *P* = 0.005; r = -0.960, *P*= 0.003; r = -0.248, *P* = 0.001; r = -0.198, *P* = 0.011) (**Table 5**). Multiple logistic regression indicated that, compared with the first quartile, the TyG index of the other three quartiles was closely associated with glucose metabolism disorders (*P*< 0.05). After adjusting for several covariates indicated in the table, the relative risk of either IGT or DM increased with the TyG quartiles (**Table 6**). These results suggest the TyG index is a reliable predictor for β-cell dysfunction in all subjects with normal or abnormal glucose metabolism.

Table 5ACorrelation coefficients between early-phase BCF indices and TyG index in subgroups with different glucose metabolic statuses.

**Table:** 

BCF		NGT (n=276)		IGR (n=323)		DM (n=315)	
	TyG	r	*P*	r	*P*	r	*P*
**Early-phase BCF indices**							
I30/I0 Ratio DI		-0.162a	0.042	-0.318f	<0.001	-0.313e	<0.001
CP30/CP0ratio DI		-0.229a	0.004	-0.412g	<0.001	-0.350e	<0.001
△I30/△G30 ratio DI		0.039b	0.625	-0.141h	0.050	-0.249j	<0.001
△CP30/△G30 ratio DI		0.036c	0.650	-0.226g	0.002	-0.260k:	<0.001
CIR30x10-2 DI		-0.034d	0.676	-0.200h	0.005	-0.383l	<0.001
Stumvoll first phase x10-2 DI		-0.106d	0.184	-0.062i	0.391	-0.162m:	0.019

A, SBP, DBP, HbA1C adjusted; b, Age, HbA1C adjusted; c, Age, HbA1C, DBP adjusted; d, Age, SBP, DBP, TC, HbA1C adjusted; e, Gender, Age, HbA1C, DBP, HDL, FCP, FINS adjusted; f, HbA1C, HDL, FINS adjusted; g, f+FCP adjusted; h, g+Age adjusted; i, Age, TC, HbA1C, FCP, FINS adjusted; j, Gender, HbA1C adjusted; k, j+FINS adjusted; l, Gender, Age, HbA1C adjusted; m, Gender, Age, HbA1c, FINS, FCP adjusted.

Table 5BCorrelation coefficients between late-phase BCF indices and TyG index in subgroups with different glucose metabolic statuses.

**Table:** 

BCF		NGT (n=276)		IGR (n=323)		DM (n=315)	
	TyG	r	*P*	r	*P*	r	*P*
**Late-phase BCF indices**							
I120/I0ratio DI		-0.180a	0.023	-0.256e	<0.001	-0.283k	<0.001
CP120/CP0ratio DI		-0.233b	0.003	-0.369f	<0.001	-0.325k	<0.001
△I120/△G120 ratio DI		0.012c	0.878	0.012g;	0.866	-0.192l	0.005
△CP120/△G120ratio DI		0.027c	0.737	-0.032h	0.4658	-0.181m	0.009
CIR120x10-2 DI		-0.045d	0.577	-0.216i	0.002	-0.374n	<0.001
Stumvoll second phase x10-2 DI		-0.166d	0.037	-0.254J	<0.001	-0.253o	<0.001

A, DBP ajusted; b, SBP, DBP, HbA1C ajusted; c, HDL ajusted; d, Gender, SBP, DBP, TC, HbA1C ajusted; e, Age ajusted; f, Age, HbA1C ajusted; g, TC ajusted; h, HbA1c ajusted; i, Age, HbA1C, HDL, FCP, FINS ajusted; j, i+TC ajusted; k, Gender,Age, DBP, HbA1C, HDL FCP, FINS ajusted; l, Gender, DBP, HbA1C, HDL ajusted; m, Age,. SBP, DBP, HbA1C, HDL, FCP, FINS ajusted; n, Gender, HbA1C, HDL ajusted; o, Age, Gender, TC, HbA1C, FCP, FINS ajusted.

**Table 6:** Polytomous logistic regression estimates for ORs of each category of TyG with different glucose statuses.

TyG	NGT (n=276)	IGR (n=323)a	DM (n=315)a
Q1 (n = 232)	reference	reference	reference
Q2 (n = 229)	reference	1.65 (0.97to 2.81)	3.04 (1.44 to 6.42)
Q3 (n = 232)	reference	1.75 (0.99to 3.09)	4.02 (1.87 to 8.64)
Q4 (n = 221)	reference	2.67 (1.34to 5.30)	6.80 (2.88to 16.08)
*P* for trend		<0.001	<0.001

A, Age, SBP, DBP, HbA1C, HDL, FINS, FCP adjusted.

## Discussion

In this study, we assessed the correlation between the TyG index and pancreatic β cell function, which revealed that the TyG index was negatively associated with the secretory function of β cells. And the negative correlation between the TyG index and β-cell function is persistent in populations with different glucose metabolic statuses. This study identified the TyG index as a simple and inexpensive surrogate marker to predict β-cell dysfunction.

T2DM is an expanding global problem induced by multiple pathogenetic factors. Insulin resistance and impaired insulin secretion remain to be two major characteristics of T2DM. And the latter is more vital for the development, progress, and insulin treatment of T2DM (29). To date, most of the approaches for predicting β-cell function are too expensive and complicated to apply in epidemiology research. Therefore, it’s urgent to identify new indicators for predicting the β-cell function. Besides hyperglycemia, T2DM patients are always accompanied by hyperlipemia. In the pancreas, nutrient overload modifies the lipid metabolism, produces advanced glycation and lipoxidation end products, and leads to β-cell dysfunction (30), suggesting a close association between the glucolipid metabolism and β-cell function. TyG index is calculated using serum FBG and TG, which has been demonstrated as a novel marker for metabolic syndrome, insulin resistance, and cardiovascular diseases (13, 31, 32). However, the relationship between the TyG index and β-cell function remains uncertain. Here, we uncovered a negative relationship between the TyG index and islet β-cell function. Namely, the higher TyG index suggests worse β-cell function. Although our results can’t directly illustrate that increased TyG causes β-cell dysfunction, it still supports the biological plausibility that hyperglycemia and hyperlipidemia are harmful to islet β-cells (33–35). Since lipid toxicity has been reported to be an independent risk factor for facilitating β-cell dysfunction, which may explain why our study suggests a negative correlation between TyG index and β-cell dysfunction in people without glucose metabolism disturbance. Furthermore, we recommended the cut-off points of 9.31 for predicting early-phase deficiency of islet β-cell function and 9.2 for predicting the late-phase β-cell function impairment respectively, which is close to the previous reports in predicting metabolic syndrome by the cut-off values of 8.8 or 9.8 for the TyG index (36, 37). In summary, our findings expand and enhance the clinical application of the TyG index in the prediction, treatment, or prognosis of T2DM.

However, our study also has some limitations. Firstly, all the indices for evaluating insulin secretion were not derived from the gold standard method, the high-glucose clamp experiment. The correlation analysis in this study was conducted between TyG and HOMA-β or indexes calculated from OGTT results. HOMA-β has been reported to be influenced by insulin resistance. Therefore, some of the indicators may not reflect the exact level of β-cell function in populations with IGT or DM. That could be the reason why we found the correlation between the TyG index and Homa-β diminished in people with abnormal glucose metabolism. Secondly, although our study adjusted multiple confounders, several residual confounding factors, for instance, diet, physical activity, and some demographic or clinical parameters we didn’t include in the analysis may affect the conclusion. Thirdly, it’s a hospital-based cross-sectional study, which may not represent the community, and can’t prove casuality. Last but not least, the population we recruited in this study is adult Chinese, which may limit the generalization and cause selection bias. Moreover, we may need to confirm the findings of our study in a validation cohort and diminish the over-fitting issue. Although there are shortcomings in this study, this’s the first time to identify the TyG index as an alternative indicator and estimate the cut-off values for predicting β-cell dysfunction.

In conclusion, we revealed an unrecognized negative association between the TyG index and islet β-cell function, which supports the plausibility that exposure to high nutrients will trigger β-cell apoptosis and dysfunction. Our study identified the TyG index as a possible novel indicator for predicting β-cell dysfunction, it may offer advantages in evaluating the β cell function in Chinese people in a simple, economical and reliable way.

## Data availability statement

The raw data supporting the conclusions of this article will be made available by the authors, without undue reservation.

## Ethics statement

The studies involving human participants were reviewed and approved by Ethical Committee of the Third Xiangya Hospital of Central South University. The patients/participants provided their written informed consent to participate in this study.

## Author contributions

ZC: collection and assembly of data, data analysis, and preparation of the first draft of the manuscript; JW: conceptualization and design, data analysis and interpretation, manuscript writing and editing, and final approval of the manuscript. All authors reviewed and approved the manuscript.

## Funding

This work was supported by the Natural Science Foundation of Hunan Province, China (Grant No. 2021JJ30037 and 2021JJ40916).

## Conflict of interest

The authors declare that the research was conducted in the absence of any commercial or financial relationships that could be construed as a potential conflict of interest.

## Publisher’s note

All claims expressed in this article are solely those of the authors and do not necessarily represent those of their affiliated organizations, or those of the publisher, the editors and the reviewers. Any product that may be evaluated in this article, or claim that may be made by its manufacturer, is not guaranteed or endorsed by the publisher.

## Supplementary material

Supplementary Table 1 AUCs of the adjusted ROC for TyG in predicting β-cell dysfunction. a: Age, Gender, SBP, DBP, HbA1C, HDL, FINS, FCP adjusted.
