# Supplementary material for: Elevated triglyceride-glucose (TyG) index predicts impaired islet β-cell function: A hospital-based cross-sectional study
Source: Front Endocrinol (Lausanne). 2022 Sep 30;13:973655. doi: 10.3389/fendo.2022.973655 (PMC9563389; doi:10.3389/fendo.2022.973655)
Supplement: Supplementary Table 1 — AUCs of the adjusted ROC for TyG in predicting β-cell dysfunction. a: Age, Gender, SBP, DBP, HbA1C, HDL, FINS, FCP adjusted. [file Table_1.docx]

| **BCF** | | **Gender** | **Age** | **SBP** | | **DBP** | | **TC** | | | **HbA1C** | **HDL** | **FCP** | | **FINS** | | | **BMI** | **LDL** | |  |
| --- | --- | --- | --- | --- | --- | --- | --- | --- | --- | --- | --- | --- | --- | --- | --- | --- | --- | --- | --- | --- | --- |
| I30/I0 Ratio DI | | ns | ns | ns | | **11.43** | | ns | | | **38.47** | ns | **11.34** | | ns | | | **11.06** | ns | |  |
| CP30/ CP0 ratio DI | | ns | ns | ns | | ns | | ns | | | **31.42** | ns | ns | | ns | | | ns | ns | |  |
| △I30/△G30 ratio DI | | ns | ns | ns | | ns | | ns | | | **66.94** | ns | ns | | ns | | | ns | ns | |  |
| △CP30/△G30 ratio DI | | ns | ns | ns | | ns | | ns | | | **59.35** | ns | **11.34** | | ns | | | ns | ns | |  |
| CIR30x10-2 DI | | ns | ns | ns | | ns | | ns | | | **78.01** | ns | ns | | ns | | | ns | ns | |  |
| Stumvoll first  phase x10-2 DI | | ns | ns | ns | | ns | | ns | | | **37.06** | ns | ns | | ns | | | **-12.63** | ns | |  |
| I120 /I0ratio DI | | ns | ns | ns | | ns | | ns | | | **21.17** | ns | ns | | ns | | | ns | ns | |  |
| CP120 /CP0 ratio DI | | ns | ns | ns | | ns | | ns | | | **25.22** | ns | ns | | ns | | | ns | ns | |  |
| △I120 /△G120 ratio DI | | ns | ns | ns | | ns | | ns | | | **58.44** | ns | ns | | ns | | | ns | ns | |  |
| △CP120/△G120 ratio DI | | ns | ns | ns | | ns | | ns | | | **48.94** | ns | ns | | ns | | | **11.03** | ns | |  |
| CIR120x10-2DI | | ns | ns | ns | | ns | | ns | | | **69.27** | ns | ns | | ns | | | ns | ns | |  |
| Stumvoll second phase x10-2 DI | | ns | ns | | ns | | ns | | ns | **55.42** | | ns | | ns | | ns | ns | | | ns | |
|  |  | |  | |  | |  | |  |  | |  | |  | |  |  | | |  | |

**Supplementary Table 1. Change of estimates (%) of covariates in Table 3.**

**Supplementary Table 2. AUCs of the adjusted ROC for TyG in predicting β-cell dysfunction.**

| **Variable** | **Adjusted AUC (95% CI)a** | ***P*-value** |
| --- | --- | --- |
| **Early-phase β-Cell dysfunction indices** |  |  |
| I30/I0 Ratio DI | 0.77 (0.73-0.80) | ＜0.001 |
| CP30/ CP0 ratio DI | 0.74 (0.71-0.78) | ＜0.001 |
| △I30/△G30 ratio DI | 0.84 (0.81-0.87) | ＜0.001 |
| △CP30/△G30 ratio DI | 0.83 (0.80-0.86) | ＜0.001 |
| CIR30x10-2 DI | 0.89 (0.87-0.92) | ＜0.001 |
| Stumvoll first phase  x10-2 DI | 0.78 (0.75-0.82) | ＜0.001 |
| **Late-phase β-Cell dysfunction indices** |  |  |
| I120 /I0ratio DI | 0.63 (0.59-0.66) | ＜0.001 |
| CP120 /CP0 ratio DI | 0.68 (0.64-0.71) | ＜0.001 |
| △I120 /△G120 ratio DI | 0.70 (0.64-0.74) | ＜0.001 |
| △CP120/△G120 ratio DI | 0.70 (0.66-0.73) | ＜0.001 |
| CIR120x10-2DI | 0.90 (0.87-0.92) | ＜0.001 |
| Stumvoll second phase  x10-2 DI | 0.82 (0.78-0.85) | ＜0.001 |

a: Age, Gender, SBP, DBP, HbA1C, HDL, FINS, FCP adjusted.
